# Diagnostic performance of six ultrasound-based risk stratification systems in thyroid follicular neoplasm: A retrospective multi-center study

**DOI:** 10.3389/fonc.2022.1013410

**Published:** 2022-10-20

**Authors:** Jingjing Yang, Yu Sun, Xingjia Li, Yueting Zhao, Xue Han, Guofang Chen, Wenbo Ding, Ruiping Li, Jianhua Wang, Fangsen Xiao, Chao Liu, Shuhang Xu

**Affiliations:** ^1^ Endocrine and Diabetes Center, Affiliated Hospital of Integrated Traditional Chinese and Western Medicine, Jiangsu Province Academy of Traditional Chinese Medicine, Nanjing University of Chinese Medicine, Nanjing, China; ^2^ Department of Endocrinology and Metabolism, The Affiliated Suqian Hospital of Xuzhou Medical University, Suqian, China; ^3^ Key Laboratory of Traditional Chinese Medicine (TCM) Syndrome and Treatment of Yingbing of State Administration of Traditional Chinese Medicine, Jiangsu Province Academy of Traditional Chinese Medicine, Nanjing, China; ^4^ Department of Ultrasound, Affiliated Hospital of Integrated Traditional Chinese and Western Medicine, Nanjing University of Chinese Medicine, Nanjing, China; ^5^ Department of Pathology, Affiliated Hospital of Integrated Traditional Chinese and Western Medicine, Nanjing University of Chinese Medicine, Nanjing, China; ^6^ Department of General Surgery, Affiliated Hospital of Integrated Traditional Chinese and Western Medicine, Nanjing University of Chinese Medicine, Nanjing, China; ^7^ Department of Endocrinology and Diabetes, The First Affiliated Hospital of Xiamen University, School of Medicine, Xiamen University, Xiamen, China

**Keywords:** Thyroid nodule, Follicular neoplasm, Thyroid Imaging Reporting and Data System, Follicular adenoma, Thyroid follicular carcinoma

## Abstract

This study aimed to compare the diagnostic performances of six commonly used ultrasound-based risk stratification systems for distinguishing follicular thyroid adenoma (FTA) from follicular thyroid carcinoma (FTC), including the American Thyroid Association Sonographic Pattern System (ATASPS), ultrasound classification systems proposed by American Association of Clinical Endocrinologists, American College of Endocrinology, and Associazione Medici Endocrinology (AACE/ACE/AME), Korean thyroid imaging reporting and data system (K-TIRADS), European Thyroid Association for the imaging reporting and data system (EU-TIRADS), American College of Radiology for the imaging reporting and data system (ACR-TIRADS), and 2020 Chinese Guidelines for Ultrasound Malignancy Risk Stratification of Thyroid Nodules (C-TIRADS). A total of 225 FTA or FTC patients were retrospectively analyzed, involving 251 thyroid nodules diagnosed by postoperative pathological examinations in three centers from January 2013 to October 2021. The diagnostic performances of six ultrasound-based risk stratification systems for distinguishing FTA from FTC were assessed by plotting the receiver operating characteristic (ROC) curves and compared at different cut-off values. A total of 205 (81.67%) cases of FTA and 46 (18.33%) cases of FTC were involved in the present study. Compared with those of FTA, FTC presented more typical ultrasound features of solid component, hypoechoic, irregular margin and sonographic halo (all *P*<0.001). There were no significant differences in ultrasound features of calcification, shape and comet-tail artifacts between cases of FTA and FTC. There was a significant difference in the category of thyroid nodules assessed by the six ultrasound-based risk stratification systems (*P*<0.001). The areas under the curve (AUCs) of ATASPS, AACE/ACE/AME, K-TIRADS, EU-TIRADS, ACR-TIRADS and C-TIRADS in distinguishing FTA from FTC were 0.645, 0.729, 0.766, 0.635, 0.783 and 0.798, respectively. Our study demonstrated that all the six ultrasound-based risk stratification systems present potential in the differential diagnosis of FTA and FTC. Specifically, C-TIRADS exerts the best diagnostic performance among the Chinese patients. ATASPS possesses a high sensitivity, while K-TIRADS possesses a high specificity in distinguishing FTA from FTC.

## Introduction

Follicular neoplasm (FN), a type of thyroid carcinoma of follicular epithelial origin that lacks the features of papillary thyroid carcinoma (PTC), has a pathology involving follicular thyroid adenoma (FTA), follicular thyroid carcinoma (FTC), follicular variant papillary thyroid carcinoma (FVPTC) and other follicular lesions (1). In addition to PTC, FTC is the most-common differentiated thyroid cancer, accounting for 10-15% of thyroid carcinomas (2). Pathological confirmation of tumor capsule invasion and/or vascular invasion in surgically resected specimen is the only diagnostic criterion for FTC. However, the fine needle aspiration cytology (FNAC) and core needle biopsy (CNB) cannot provide a panoramic view of the entire fibrous capsule and vascular invasion, thus restricting their application in the diagnosis of FN (3, 4). Preoperative differential diagnosis of benign and malignant FN remains challenging in clinical practice.

Thyroid ultrasound is a preferred tool for thyroid nodule examination. A growing number of thyroid nodules have been detected by ultrasonography. To standardize the evaluation of malignant thyroid nodules, various clinical societies have developed ultrasound-based systems to stratify malignant risks (5). Based on the Thyroid Imaging Reporting and Data System (TIRADS) proposed by Horvath et al. (6), several “pattern-based” systems and “score-based” systems have been established. The former includes the ATASPS (American Thyroid Association Sonographic Pattern System), K-TIRADS (Korean Society of Thyroid Radiology), AACE/ACE/AME (American College of Endocrinology, and Associazione Medici Endocrinologi Medical), K-TIRADS (Korean Society of Thyroid Radiology), and EU-TIRADS (European Thyroid Association). The latter is represented by ACR-TIRADS (American College of Radiology) and C-TIRADS (2020 Chinese Guidelines for Ultrasound Malignancy Risk Stratification of Thyroid Nodules). Meanwhile, contrast-enhanced ultrasound (CEUS) has been introduced to evaluate thyroid parenchyma, but it is debatable whether CEUS can improve the diagnostic accuracy of ultrasound imaging reporting systems at present (7). The accuracy of artificial intelligence tools in characterizing thyroid nodules and cancers remains controversial (8). Therefore, ultrasound risk stratification systems are still the main tool for thyroid nodule examination.

The ultrasound characteristics suspected by the abovementioned systems are related to PTC, including the solid component, hypoechoic appearance, irregular margin, microcalcification, and taller-than-wide (9). Ultrasound findings of hypoechoic appearance, punctate microcalcification, indistinct or irregular margin, taller-than-wide, and increased intranodular blood flow may help establish the diagnosis of FTC (10). However, ultrasound characteristics of FTC and FTA may substantially overlap, typically manifested as a solitary, smooth margin, homogeneously isechoic or hypoechoic nodule with a peripheral halo, parallel orientation to the skin surface, and no lymph node enlargement (1). In addition, there is a significant difference in the incidence between PTC and FTC. The Surveillance, Epidemiology, and Results Program (SEER) data from 1974 to 2013 revealed that the incidence of PTC and FTC increased by an average of 4.4% and 0.6% per year, respectively (11). Trimboli et al. (12) showed that the vast majority (88.9%-99.6%) of malignant tumor specimens reported by the ultrasound-based risk stratification system were diagnosed as PTC. Therefore, whether the existing ultrasound-based risk stratification systems are suitable for the diagnosis of FN remains controversial, and current clinical data on their diagnostic potential are inconsistent (13–15).

The present multi-center retrospective study aimed to compare the diagnostic performances of ATASPS, AACE/ACE/AME, K-TIRADS, EU-TIRADS, ACR-TIRADS and C-TIRADS in distinguishing FTA from FTC, thus providing references for preoperative diagnosis of FN.

## Materials and methods

### Subjects

**Figure 1:**
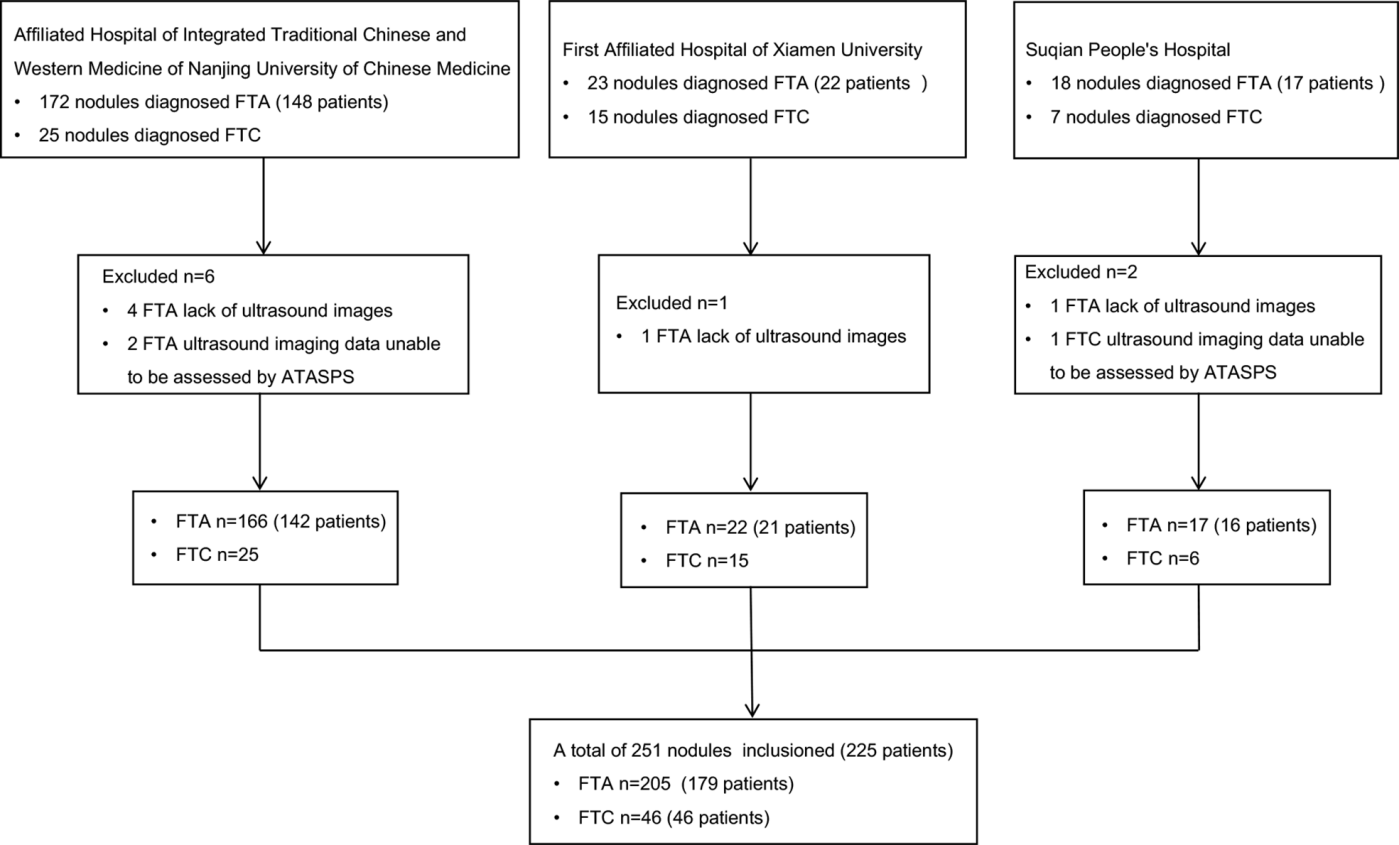
Flowchart summarizing the patient inclusion process.

A total of 225 FTA or FTC patients postoperatively diagnosed in the Affiliated Hospital of Integrated Traditional Chinese and Western Medicine of Nanjing University of Chinese Medicine (Nanjing, China), the First Affiliated Hospital of Xiamen University (Xiamen, China), and Suqian People’s Hospital (Suqian, China) from January 2013 to October 2021 were retrospectively analyzed, based on their clinical data, thyroid ultrasound reports and postoperative pathological data (**Figure 1**).

Exclusion criteria: (i) Clinical data were incomplete; ultrasound elasticity imaging data or postoperative pathological data were unable to be assessed by ATASPS, AACE/ACE/AME, K-TIRADS, EU-TIRADS, ACR-TIRADS and C-TIRADS; (ii) Pathological results were inconsistent with clinical data or ultrasound results.

### Ultrasound examination

Ultrasonography examinations were performed by three sonographers in three centers equipped with using the Hi Vision Preirus ultrasound machine. All sonographers had more than 5 years of experience in superficial organ ultrasound diagnosis, and were specialized in differential diagnosis of thyroid diseases. Thyroid nodules were assessed based on the following ultrasound features: maximum diameter (cm); component (solid, mixed solid and cystic, or cystic); echogenicity (hyperechoic, isoechoic, hypoechoic, or markedly hypoechoic); margin (smooth, or irregular); calcification (absent, microcalcification, macrocalcification, or peripheral calcification); shape (wider-than-tall or taller-than-wide); presence of halo and comet-tail artifacts; suspected invasion of neck lymph nodes and extrathyroid invasion.

All thyroid nodules were retrospectively assessed by the ATASPS, AACE/ACE/AME, K-TIRADS, EU-TIRADS, ACR-TIRADS and C-TIRADS (5, 16–20). Based on the conventional assessment, the former four classified thyroid nodules into the following categories: benign, very low suspicion or low suspicion, intermediate suspicion and high suspicion (5, 16, 18, 20). ACR-TIRADS and C-TIRADS assessed thyroid nodules by grading the typical ultrasound characteristics and calculating the total scores (17, 19). Notably, a total of 13 cases of FTA and 9 cases of FTC, which were assessed by ATASPS, did not belong to any category.

### Statistical analysis

Continuous measurement data that were normally distributed (e.g., age, diameter of thyroid nodules) were expressed as $\bar{x}$ s, and compared by the paired *t*-test. Enumeration data (e.g., sex, ultrasound characteristics, thyroid nodule category) were expressed as percentage, and compared by the Chi-square test. ROC curves were plotted with the sensitivity and specificity as the ordinate and abscissa, respectively, in which the postoperative pathology served as the gold standard. The AUC was calculated based on the binomial distribution of the category of thyroid nodules assessed by the six systems. Moreover, the Youden index, cut-off value, sensitivity, specificity, positive predictive value (PPV) and negative predictive value (NPV) were calculated. *P*<0.05 was considered as statistically significant.

## Results

### Baseline characteristics

A total of 225 patients (251 thyroid nodules) were included in the present study, including 179 FTA patients (205 nodules) and 46 FTC patients (46 nodules). There were 42 male and 137 female FTA patients, and 12 male and 34 female FTC patients. No significant differences in the age and the maximum diameter of thyroid nodules were detected between FTA and FTC patients (**Table 1**). The ratio of FTA or FTC in females was significantly higher than that in males.

**Table 1:** Baseline characteristics of FTA and FTC patients.

	Pathology of thyroid nodules		Total	*P* value
	FTA	FTC	
Thyroid nodules (n, %)	205 (81.67)	46 (18.33)	251	
Case number (n, %)	179 (79.56)	46 (20.44)	225	
Age (years)	47.99 ± 13.63	48.20 ± 16.25		0.932
Sex (n, %)				
Male	42 (23.46)	12 (26.09)		
Female	137 (76.54)	34 (73.91)		
Thyroid nodule diameter (cm)	3.27 ± 1.66	3.30 ± 1.71		0.896

FTA, follicular thyroid adenoma; FTC, follicular thyroid carcinoma.

### Ultrasound characteristics and malignancy rates of thyroid nodules

**Figure 2:**
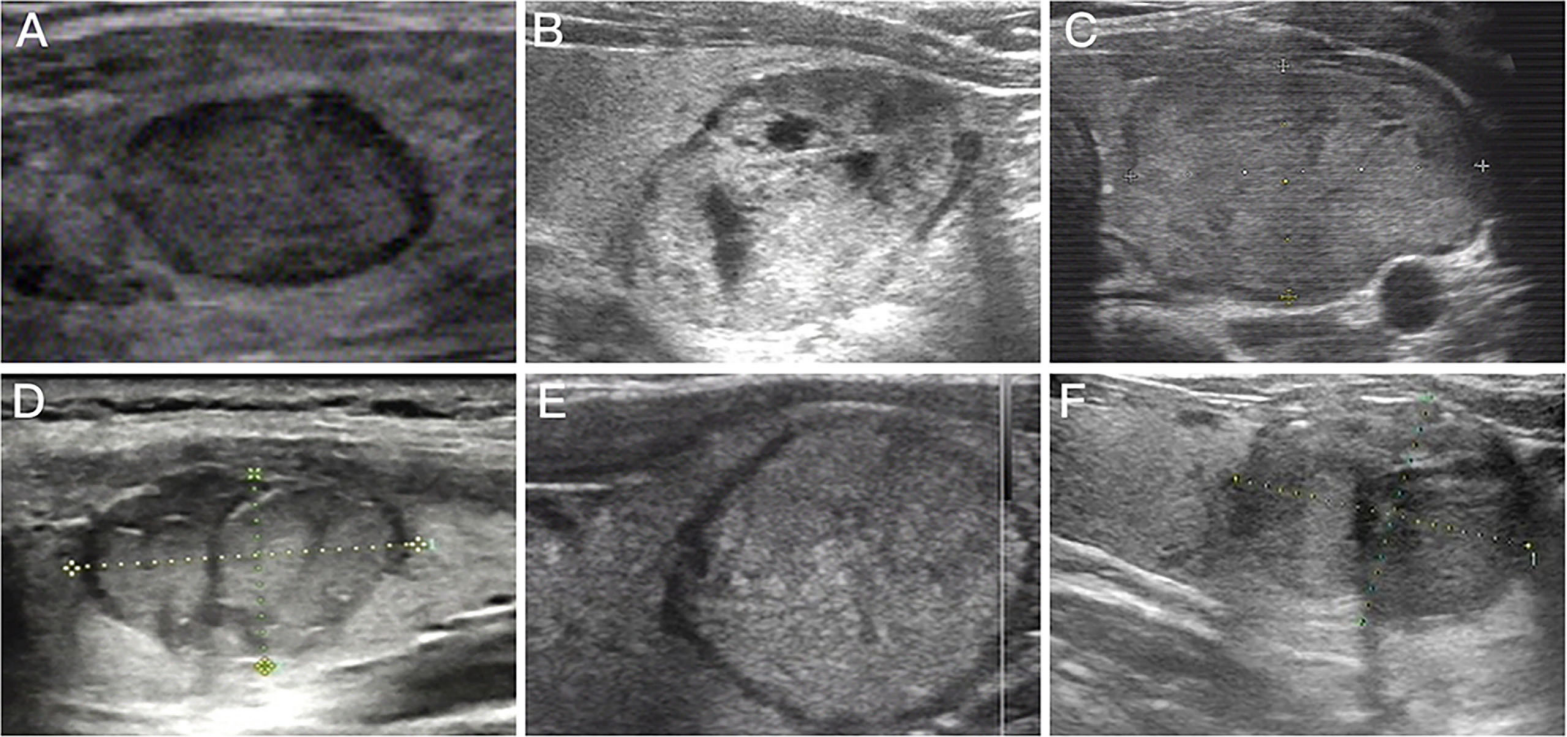
Preoperative ultrasound images for follicular thyroid adenoma and follicular thyroid carcinoma. **(A)** Preoperative ultrasound image of nodule with regular margin diagnosed as follicular thyroid adenoma; **(B)** Mixed nodule that postoperative pathological diagnosis was follicular thyroid adenoma; **(C)** Preoperative ultrasound image of isoechoic nodule diagnosed as follicular thyroid adenoma; **(D)** Preoperative ultrasound image of nodule with irregular margin diagnosed as follicular thyroid carcinoma; **(E)** Purely solid nodule that postoperative pathological diagnosis was follicular thyroid carcinoma; **(F)** Preoperative ultrasound image of hypoechoic nodule diagnosed as follicular thyroid carcinoma.

Most of included thyroid nodules presented typical ultrasound characteristics of solid components (44.22%), hyperechoic/isoechoic appearance (80.88%), regular margin (83.67%), non-calcification (88.44%) and wider-than-tall (98.41%) (**Table 2**). The incidences of solid (34.14% vs. 89.13%, *P*<0.001), hypoechoic (16.10% vs. 30.44%, *P=*0.007), irregular margin (4.88% vs. 28.26%, *P*<0.001) and presence of halo (15.61% vs. 36.96%, *P*<0.001) in cases of FTA were significantly lower than those in cases of FTC (**Figure 2**). No significant differences in the incidences of calcification (*P=*0.936), shape of thyroid nodules (*P=*0.099) and the presence of comet-tail artifacts (*P=*0.915) were found between FTA and FTC.

**Table 2:** Ultrasound characteristics of FTA and FTC, and the malignancy rate.

Ultrasound characteristics	Pathology		Total	Malignancy rate (%)	*P* value
	FTA n=205	FTC n=46		
Component					<0.001
Solid	70 (34.14)	41 (89.13)	111 (44.22)	36.94	
Cystic	83 (40.49)	0	83 (33.07)	0	
Mixed solid and cystic	52 (25.37)	5 (10.87)	57 (22.71)	8.77	
Echogenicity					0.007
Markedly hypoechoic	0 (0)	1 (2.17)	1 (0.40)	100	
Hypoechoic	33 (16.10)	14 (30.44)	47 (18.73)	29.78	
Isoechoic/hyperechoic	172 (83.90)	31 (67.39)	203 (80.88)	15.27	
Margin					<0.001
Irregular	10 (4.88)	13 (28.26)	208 (82.87)	6.25	
Regular	195 (95.12)	33 (71.74)	43 (17.13)	76.74	
Calcification					0.936
Microcalcification	11 (5.37)	3 (6.52)	14 (5.58)	21.43	
Macrocalcification	12 (5.85)	3 (6.52)	15 (5.98)	20.00	
Absent	182 (88.78)	40 (86.96)	222 (88.44)	18.01	
Shape					0.099
Taller-than-wide	2 (0.6)	2 (4.35)	4 (1.59)	50.00	
Wider-than-tall	203 (99.4)	44 (95.65)	247 (98.41)	17.81	
Comet-tail artifact					
Detected	5 (2.44)	1 (2.17)	6 (2.41)	16.67	0.915
Not detected	200 (97.56)	45 (97.82)	245 (97.59)	18.37	
Halo					<0.001
Detected	32 (15.61)	17 (36.96)	49 (19.52)	34.69	
Not detected	173 (84.39)	29 (63.04)	202 (80.48)	14.36	

FTA, follicular thyroid adenoma; FTC, follicular thyroid carcinoma.

The malignancy rate of all solid thyroid nodules (36.94%) was significantly higher than that of predominately solid (7.01%) or predominately cystic ones (1.75%), and that of markedly hypoechoic (100%) or hypoechoic thyroid nodules (29.78%) was significantly higher than that of hyperechoic/isoechoic nodules (15.27%). Thyroid nodules with irregular margins showed a significantly high malignancy rate than those with regular margins (76.74% vs. 6.25%). The malignancy rate of thyroid nodules with tall-than-wider shape was significantly higher than that of the remaining (50.00% vs. 17.81%). Moreover, a significantly higher malignancy rate was detected in thyroid nodules with halos than in those lacking halos (34.69% vs. 14.36%).

### Malignancy rates of thyroid nodules categorized by ultrasound-based risk stratification systems

There was a significant difference in the category of thyroid nodules assessed by the six ultrasound-based risk stratification systems (all *P*<0.001, **Table 3**). In detail, 40.49%, 42.44%, 43.90%, 79.20%, 56.59% and 49.27% of FTA were considered as benign, moderately suspicious, K-TR3, EU-TR3, ACR-TR2 and C-TR3 assessed by ATASPS, AACE/ACE/AME, K-TIRADS, EU-TIRADS, ACR-TIRADS and C-TIRADS, respectively. Among them, the highest malignancy rate was detected in thyroid nodules with K-TR5 (66.67%), followed by C-TR4C (55.56%).

**Table 3:** Malignancy rate of thyroid nodules assessed by the six ultrasound-based risk stratification systems.

	Pathology		Malignancy rate (%)	
	Benign thyroid nodules n=205	Malignant thyroid nodules n=46		*P* value
ATASPS				<0.001
Benign	83 (40.49)	1 (2.17)	1.19	
Very low suspicion	43 (20.98)	1 (2.17)	2.27	
Low suspicion	48 (23.41)	22 (47.83)	31.43	
Intermediate suspicion	15 (7.32)	10 (21.74)	40	
High suspicion	3 (1.46)	3 (6.52)	50	
Nonclassifiable group	13 (6.34)	9 (19.57)	40.9	
AACE/ACE/AME				<0.001
Low	76 (37.07)	0	0	
Intermediate	87 (42.44)	24 (52.17)	21.62	
High suspicion	42 (20.49)	22 (47.82)	34.38	
K-TIRADS				<0.001
Benign (K-TR2)	81 (39.51)	0	0	
Low suspicion (K-TR3)	90 (43.90)	26 (56.52)	22.41	
Intermediate suspicion (K-TR4)	30 (14.63)	12 (26.09)	28.57	
High suspicion (K-TR5)	4 (1.95)	8 (17.39)	66.67	
EU-TIRADS				
Benign (EU-TR2)	0	0	0	<0.001
Low risk (EU-TR3)	162 (79.02)	24 (52.18)	12.9	
Intermediate risk (EU-TR4)	22 (10.73)	6 (13.04)	21.43	
High risk (EU-TR5)	21 (10.25)	16 (34.78)	43.24	
ACR-TIRADS				
Benign (ACR-TR1)	0	0	0	<0.001
Not suspicious (ACR-TR2)	116 (56.59)	2 (4.35)	1.7	
Mildly suspicious (ACR-TR3)	45 (21.95)	23 (50.00)	33.82	
Moderately suspicious (ACR-TR4)	37 (18.05)	15 (32.61)	28.85	
Highly suspicious (ACR-TR5)	7 (3.41)	6 (13.04)	46.15	
C-TIRADS				<0.001
C-TR2	0	0	0	
C-TR3	101 (49.27)	4 (8.69)	3.81	
C-TR4A	72 (35.12)	22 (47.83)	23.4	
C-TR4B	24 (11.71)	10 (21.74)	29.41	
C-TR4C	8 (3.90)	10 (21.74)	55.56	
C-TR5	0	0	0	

FTA, follicular thyroid adenoma; FTC, follicular thyroid carcinoma; 2 ATASPS, The American Thyroid Association Sonographic Pattern System; AACE/ACE/AME, American Association of Clinical Endocrinologists, American College of Endocrinology, and Associazione Medici Endocrinology; K-TIRADS, Korean thyroid imaging reporting and data system; EU-TIRADS, European Thyroid Association for the imaging reporting and data system; ACR-TIRADS, American College of Radiology for the imaging reporting and data system; C-TIRADS, 2020 Chinese Guidelines for Ultrasound Malignancy Risk Stratification of Thyroid Nodules.

### Diagnostic performances of six ultrasound-based risk stratification systems in distinguishing FTA from FTC

**Figure 3:**
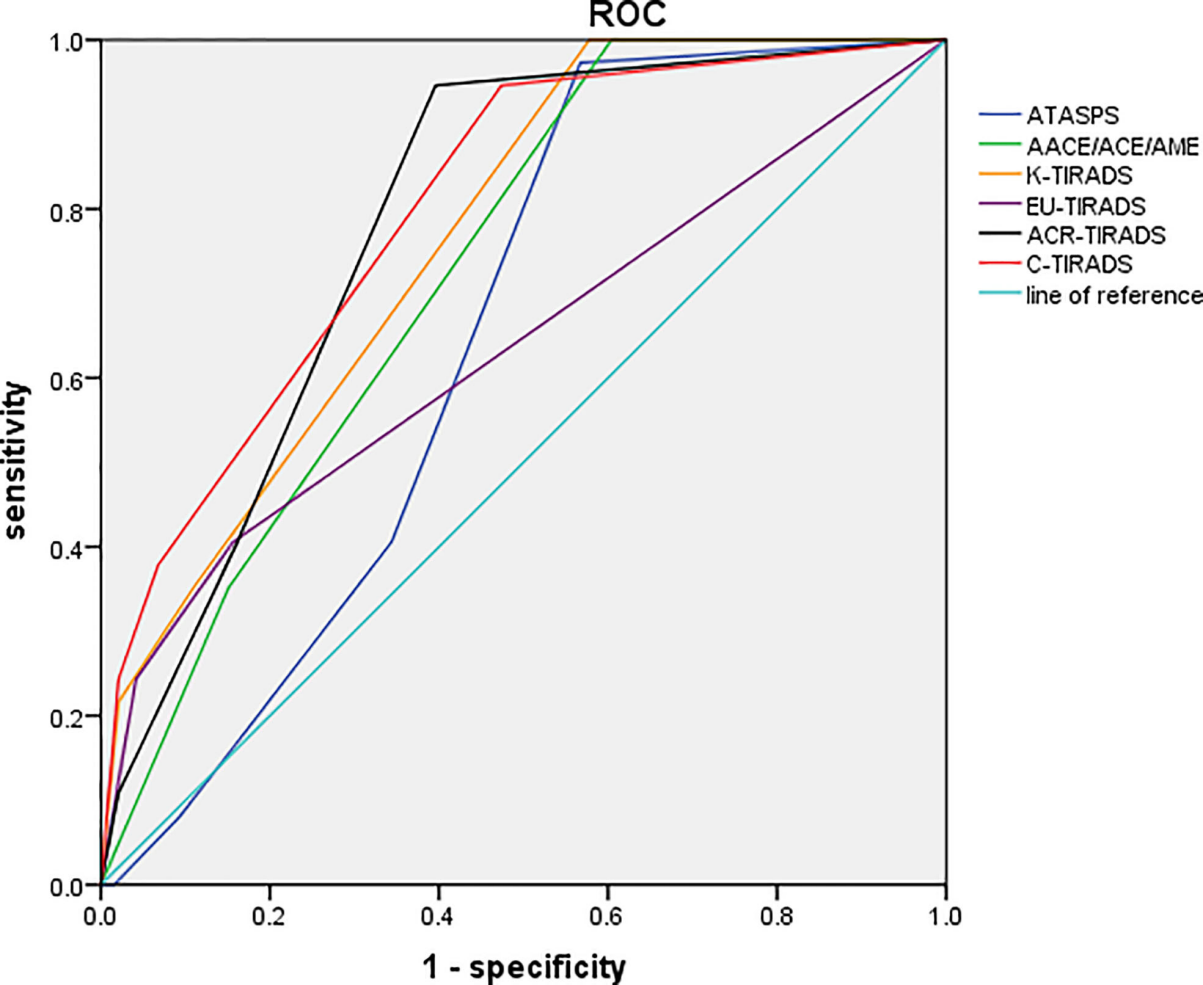
ROC curves of the six ultrasound-based risk stratification systems for distinguishing FTA from FTC. ROC, receiver operating characteristic; FTA, follicular thyroid adenoma; FTC, follicular thyroid carcinoma; ATASPS, The American Thyroid Association Sonographic Pattern System; AACE/ACE/AME, American Association of Clinical Endocrinologists, American College of Endocrinology, and Associazione Medici Endocrinology; K-TIRADS, Korean thyroid imaging reporting and data system; EU-TIRADS, European Thyroid Association for the imaging reporting and data system; ACR-TIRADS, American College of Radiology for the imaging reporting and data system; C-TIRADS, 2020 Chinese Guidelines for Ultrasound Malignancy Risk Stratification of Thyroid Nodules.

The diagnostic performances of six ultrasound-based risk stratification systems for distinguishing FTA from FTC were assessed by plotting the ROC curves. The AUCs of ATASPS, AACE/ACE/AME, K-TIRADS, EU-TIRADS, ACR-TIRADS and C-TIRADS in distinguishing FTA from FTC were 0.645, 0.729, 0.766, 0.635, 0.783 and 0.798, respectively (**Figure 3**, all *P*<0.05). Based on the Youden index, the optimal cut-off of ATASPS, AACE/ACE/AME, K-TIRADS, EU-TIRADS, ACR-TIRADS and C-TIRADS in distinguishing FTA from FTC was low suspicion, intermediate-risk, K-TR4, EU-TR5, ACR-TR3 and C-TR4A, respectively. In particular, the largest AUC was detected in C-TIRADS (0.798; 95%CI, 0.743-0.862), with sensitivity, specificity, PPV and NPV of 94.59% (95%CI, 81.85-99.34), 52.62% (95%CI, 45.38-59.81), 27.86% (95%CI, 24.59-31.36) and 98.17% (95%CI, 92.24-99.57), respectively. No significant difference in AUC was detected among the six ultrasound-based risk stratification systems. The highest sensitivity and specificity were achieved by the ATASPS (97.30%; 95%CI, 85.89-99.98) and K-TIRADS (97.92%; 95%CI, 94.81-99.48), respectively (**Table 4**).

**Table 4:** The diagnostic performance of 2015 ATA, AACE/ACE/AME, K-TIRADS, EU-TIRADS, ACR-TIRADS and C-TIRADS.

	Cut-off	Sensitivity(%, 95%CI)	Specificity(%, 95%CI)	PPV(%, 95%CI)	NPV(%, 95%CI)	AUC
ATASPS	Low suspicion	97.30 (85.89-99.98)	43.23 (36.17-50.64)	24.81 (22.49-27.46)	98.83 (92.34-99.82)	0.645 (0.573-0.716)
AACE/ACE/AME	Intermediate suspicion	93.85 (91.73-99.91)	39.58 (32.64-46.91)	24.28 (22.18-26.35)	99.31 (95.21-99.98)	0.729 (0.667-0.790)
K-TIRADS	K-TR4	21.62 (9.29-38.27)	97.92 (94.81-99.48)	66.73 (38.82-86.37)	86.69 (84.59-88.53)	0.766 (0.707-0825)
EU-TIRADS	EU-TR5	24.32 (11.82-41.25)	95.83 (92.01-98.27)	52.93 (31.77-73.24)	86.83 (84.51-88.86)	0.635 (0.547-0.723)
ACR-TIRADS	ACR-TR3	94.59 (81.85-99.34)	60.42 (53.18-67.42)	31.55 (27.63-35.89)	98.32 (93.76-99.65)	0.783 (0.702-0.847)
C-TIRADS	C-TR4A	94.59 (81.85-99.34)	52.62 (45.38-59.81)	27.86 (24.59-31.36)	98.17 (92.24-99.57)	0.798 (0.743-0.862)

CI, confidence interval; PPV, positive predictive value; NPV, negative predictive value; AUC, area under the curve; 2015 ATA, the 2015 American Thyroid Association management guidelines for adult patients with thyroid nodules and differentiated thyroid cancer; AACE/ACE/AME, American Association of Clinical Endocrinologists, American College of Endocrinology, and Associazione Medici Endocrinology; K-TIRADS, Korean thyroid imaging reporting and data system; EU-TIRADS, European Thyroid Association for the imaging reporting and data system; ACR-TIRADS, American College of Radiology for the imaging reporting and data system; C-TIRADS, 2020 Chinese Guidelines for Ultrasound Malignancy Risk Stratification of Thyroid Nodules.

## Discussion

The diagnosis of FTC depends upon pathological confirmation of tumor capsular invasion and/or vascular invasion. Nevertheless, conventional tools for assessing thyroid nodules like ultrasonography, FNAC and CNB are unable to visualize these invasions, thereby discounting their diagnostic potential (21). Therefore, preoperative differential diagnosis of benign and malignant FN remains challenging. It is reported that the incidences of FTC and Hürthle cell carcinoma (HHC) from 1974 to 2013 remained stable (0.5-0.6% and 1.1-1.6%, respectively) in males and females, or even presented a decreasing trend (22). Englum et al. (23) have demonstrated that the male gender, black people, tumor size increase and distant metastasis are predictive factors for the diagnosis of FTC. In addition, as patients’ age increased from 45 years, patients were more likely to be diagnosed with FTC. In the present study, there were no significant differences in the sex, mean age and thyroid nodule size between FTC and FTA patients. Therefore, the potential of age and sex in predicting benign or malignant FN remains to be further analyzed.

High-resolution ultrasonography of thyroid nodules is of great significance in the screening, diagnosis, preoperative evaluation, and postoperative follow-up (24, 25). In the present study, we compared the ultrasound characteristics of FTA and FTC patients, involving 251 thyroid nodules. Only 5 FTC were mixed cystic and solid thyroid nodules, and most of the rest only were solid nodules; 34.13% and 40.49% cases of FTA were solid and cystic, respectively, and the remaining were mixed solid and cystic; 30.73% of FTA cases were postoperatively diagnosed as follicular adenoma with cystic lesions or hemorrhage. Mixed cystic and solid thyroid nodules are mainly caused by the degeneration of benign thyroid nodules, including cystic degeneration, hemorrhage, necrosis, etc. Only a small number of thyroid nodules contain epithelial tissues, and the malignancy rate of mixed cystic and solid thyroid nodules ranges 5.4-11.1% (26, 27). FTC is solid in most cases, closely linked with the angiogenesis during the process of tumor cell formation and growth. It is reported that the vascular endothelial growth factor-2 (VEGFR2) signaling pathway acts to promote the pathological angiogenesis during tumor cell generation and hyperplasia (28). Asghar et al. (29) have revealed that stromal interaction molecule 1 (STIM1) is significantly upregulated in thyroid tumor tissues than in normal thyroid tissues, the expression level of which is higher in FTC than in PTC. Moreover, knockdown of STIM1 results in the downregulation of VEGFR2 in FTC cells *in vitro*.

Compared with FTA, FTC mainly manifested the following ultrasound characteristics, including solid component, hypoechoic appearance, irregular margin and the presence of halo. Sillery et al. (30) have demonstrated that the sonographic features of FTA are similar to those of FTC, but larger lesion size, lack of a sonographic halo, hypoechoic, and absence of cystic change are conducive to the diagnosis of FTC. The EU-TIRADS proposes that interrupted peripheral macrocalcifications, a thick halo, or lack of a halo would increase the malignancy risk, while a thin halo indicates a benign thyroid nodule (18). Li et al. (31) have suggested that an intermittent or uninterrupted irregular halo, hypoechoic or markedly hypoechoic, and solid component are independent risk factors for FTC. In the present study, there were 30 and 2 cases of FTA with a thin and a thick halo, respectively, while 14 and 3 cases of FTC presented a thin and a thick halo, respectively. No significant difference in the incidence of thin/thick halo was detected between FTA and FTC patients (*P*=0.326). Collectively, ultrasound characteristics of FTC were mainly characterized as solid component, hypoechoic appearance, and irregular margin. Moreover, the ultrasound characteristics of a sonographic halo should be further analyzed.

Microcalcification used to be considered as a classical sign of malignant thyroid tumors, while coarse calcification or macrocalcification is more commonly detected in benign nodules. Kuo et al. (32) have suggested that calcification on the ultrasound image is an independent factor for predicting FTC. Therefore, it is believed that calcification contributes to distinguishing FTC from FTA. Our study showed that the malignancy rate of thyroid nodules with microcalcification was slightly higher than those with macrocalcification (21.43% vs. 18.01%), while no significant difference in the calcification type was detected between FTA and FTC. A total of 11 cases of cystic or mixed solid and cystic FTA presented microcalcification, and among them, 8 cases represented the comet-tail artifacts. However, comet-tail artifacts were not detected in 3 cases of FTC with solid nodules. Hyperechoic along with comet-tail artifacts in thyroid nodules with cystic components are highly suggestive of benignity (5, 33). In addition to the calcification, echogenic foci also suggest the concentrated colloid, which is the manifestation of benign cystic thyroid nodules. Notably, echogenic foci with comet-tail artifacts are not the absolute predictor of benign thyroid nodules. Wu et al. (34) have argued that echogenic foci with comet-tail artifacts in cystic components are the predictor of benign thyroid nodules, while those in solid components are not an absolute predictor of benign thyroid nodules. Therefore, punctate echogenic foci with comet-tail artifacts contribute to distinguishing benign thyroid nodules from malignant ones. The diagnostic potential of microcalcification in FTC, however, remains unclear.

Based on the Youden index, the optimal cut-off values of ATASPS, AACE/ACE/AME, K-TIRADS, EU-TIRADS, ACR-TIRADS and C-TIRADS in distinguishing FTA from FTC were low suspicion pattern, moderately suspicious, K-TR4, EU-TR5, ACR-TR3 and C-TR4A, respectively. Castellana et al. (14) have categorized 45 cases of FTC using 7 ultrasound-based risk stratification systems. When they were classified in 7 US RSSs, the prevalent classes were intermediate risk by AACE/ACE/AME (53%), TR4 by ACR-TIRADS (60%), U4 by BTA (50%), K-TIRADS 4 by K-TIRADS (53%) and TIRADS 4A by TIRADS (75%). Moreover, AACE/ACE/AME, ACR-TIRADS, ATA, EU-TIRADS and TIRADS missed 1 case of FTC (16%) and K-TIRADS did not miss any case based on the cut-off value of moderate suspicion. Our data showed that the specificity of K-TIRADS in diagnosing FTC was remarkably higher than that of other systems.

Existing data on the evaluation of FN using different ultrasound-based risk stratification systems are inconsistent. Here, the AUCs of diagnosing FTC by the six ultrasound-based risk stratification systems ranged from 0.635 to 0.798 (*P*<0.05). Lin et al. (15) have reported that the AUC of K-TIRADS, EU-TIRADS, ACR-TIRADS, C-TIRADS, ACEE and ATA in diagnosing FTC based on the cut-off value of moderate or highly suspicion is disappointing (AUC=0.511-0.611, *P*<0.05). Liu et al. (35) have revealed the acceptable performance of ATA (AUC=0.744, *P*<0.001) and ACR-TIRADS (AUC=0.744, *P*<0.001) in distinguishing benign FN from malignant ones. Hamour et al. (36) have shown that the standardized use of TI-RADS and educational initiatives increases the clinical value of TI-RADS. Notably, most cases of FTC can be preoperatively identified by current ultrasound-based risk stratification systems and subjected to FNAC, because the lesion size is considered as the indicator for FNAC (14). Given that some cases of FTC are non-highly suspicious and difficult to be identified by cytological evaluation, follow-up ultrasonography is recommended for thyroid nodules with uncertain cytological findings.

Several limitations in this study should be noted. First of all, it was a retrospective study involving surgically treated patients after thyroid lobectomy or total thyroidectomy, which may result in the selection bias and increased malignancy rate of thyroid nodules. Second, it was a multi-center study that may cause differences related to investigators at different institutions. Prospective studies with a larger sample size are needed to analyze other suspicious factors for diagnosing malignant thyroid nodules in the future.

Taken together, all the six ultrasound-based risk stratification systems display favorable diagnostic potential for FN. Among them, C-TIRAD presents the best diagnostic performance, followed by ACR-TIRADS, K-TIRADS, AACE/ACE/AME and ATASPS. In addition, ATASPS and K-TIRADS pose the highest sensitivity and specificity in distinguishing FTA from FTC, respectively.

## Data availability statement

The raw data supporting the conclusions of this article will be made available by the authors, without undue reservation.

## Ethics statement

The studies involving human participants were reviewed and approved by the ethics committee of the Jiangsu Province Academy of Traditional Chinese Medicine. Written informed consent for participation was not required for this study in accordance with the national legislation and the institutional requirements.

## Author contributions

JY, FX, and SX developed the research questionnaire and wrote the protocol for this study. JY, FX, and YS were responsible for data collection and analysis. XL, GC, WD, and RL participated the diagnosis. JW were the operators for surgery. YZ and XH were responsible for the perioperative management. SX, FX, and CL interpreted the results. JY and YS wrote the article. SX and CL revised it critically for important intellectual content. All authors agreed to take responsibility for the integrity of the data and the accuracy of the data analysis. All authors contributed to the article and approved the submitted version.

## Funding

This work was granted by the Key Research and Development Plan (Social Development) of Jiangsu Province, BE2020726, Medical Scientific Research Foundation of Jiangsu Province of China (Surface project), M2020102, Research and Practice Innovation Project of Nanjing University of Chinese Medicine, SJCX21_0752.

## Conflict of interest

The authors declare that the research was conducted in the absence of any commercial or financial relationships that could be construed as a potential conflict of interest.

## Publisher’s note

All claims expressed in this article are solely those of the authors and do not necessarily represent those of their affiliated organizations, or those of the publisher, the editors and the reviewers. Any product that may be evaluated in this article, or claim that may be made by its manufacturer, is not guaranteed or endorsed by the publisher.
